# T Cell Receptor Signaling Can Directly Enhance the Avidity of CD28 Ligand Binding

**DOI:** 10.1371/journal.pone.0089263

**Published:** 2014-02-24

**Authors:** Mariano Sanchez-Lockhart, Ana V. Rojas, Margaret M. Fettis, Richard Bauserman, Trissha R. Higa, Hongyu Miao, Richard E. Waugh, Jim Miller

**Affiliations:** 1 David H Smith Center for Vaccine Biology and Immunology and Department of Microbiology and Immunology, University of Rochester, Rochester, New York, United States of America; 2 Department of Biostatistics and Computational Biology, University of Rochester, Rochester, New York, United States of America; 3 Department of Biomedical Engineering, University of Rochester, Rochester, New York, United States of America; University of Iowa, United States of America

## Abstract

T cell activation takes place in the context of a spatial and kinetic reorganization of cell surface proteins and signaling molecules at the contact site with an antigen presenting cell, termed the immunological synapse. Coordination of the activation, recruitment, and signaling from T cell receptor (TCR) in conjunction with adhesion and costimulatory receptors regulates both the initiation and duration of signaling that is required for T cell activation. The costimulatory receptor, CD28, is an essential signaling molecule that determines the quality and quantity of T cell immune responses. Although the functional consequences of CD28 engagement are well described, the molecular mechanisms that regulate CD28 function are largely unknown. Using a micropipet adhesion frequency assay, we show that TCR signaling enhances the direct binding between CD28 and its ligand, CD80. Although CD28 is expressed as a homodimer, soluble recombinant CD28 can only bind ligand monovalently. Our data suggest that the increase in CD28-CD28 binding is mediated through a change in CD28 valency. Molecular dynamic simulations and *in vitro* mutagenesis indicate that mutations at the base of the CD28 homodimer interface, distal to the ligand-binding site, can induce a change in the orientation of the dimer that allows for bivalent ligand binding. When expressed in T cells, this mutation allows for high avidity CD28–CD80 interactions without TCR signaling. Molecular dynamic simulations also suggest that wild type CD28 can stably adopt a bivalent conformation. These results support a model whereby inside-out signaling from the TCR can enhance CD28 ligand interactions by inducing a change in the CD28 dimer interface to allow for bivalent ligand binding and ultimately the transduction of CD28 costimulatory signals that are required for T cell activation.

## Introduction

Efficient T cell activation requires co-ligation of the T cell antigen receptor (TCR) and costimulatory receptors. T cell encounter with antigen in the absence of costimulation leads to limited T cell activation and can induce anergy. The best described costimulatory molecule is CD28. CD28 has been shown to have a broad impact on many aspects of T cell function, including T cell activation, elaboration of effector cytokine expression, enhanced expansion and survival, upregulation of metabolic activity, effector cell differentiation, memory responses, and tolerance [1], [2], [3], [4], [5]. Interestingly, one of the key consequences of the innate immune response to microbial challenge is the upregulation on dendritic cells of CD80 and CD86, the ligands for CD28. Thus, CD28 can be viewed as the T cell-associated receptor for detection of pathogens.

CD28 functions as a modifier and amplifier of TCR-derived signals [6]. T cell activation takes place in the context of an immunological synapse that is formed at the cell:cell contact between a T cell and an antigen presenting cell (APC). T cell signaling is initiated and sustained by the formation of TCR microclusters that form at the periphery of the contact site and move to and coalesce within the center of the immunological synapse [7]. TCR and CD28 are rapidly colocalized in these microclusters [8]. Although the precise mechanisms of CD28 costimulation are not fully understood, CD28 functions in part through direct amplification of the TCR signal, for example though activation of PI3K and Lck [6], and through unique contributions, notably the recruitment of PKCθ [9], [10], [11], [12], [13].

In addition to the well-described effects of CD28 on modulating TCR signaling, recent studies indicate that TCR can also modulate CD28 function. TCR signaling can induce a rapid reorientation of the cytosolic tail domains within the CD28 homodimer as detected by a change in fluorescence resonance energy transfer (FRET) [14]. TCR signaling is also necessary for sustained localization of CD28 to the immunological synapse [11], [13] and TCR signaling can induce CD28 polarization toward CD80-positive cells [14]. These results raise the possibility that TCR signaling may be able to enhance CD28 localization at the immunological synapse by regulating the ability of CD28 to interact with ligand. This process of inside-out signaling, whereby intracellular signaling from one receptor can enhance ligand binding of a second receptor, is well established for integrins [15], [16]. Inside-out signaling for integrins is initiated by a conformational change in the cytosolic domains that is transduced across the plasma membrane and results in a dramatic conformational change in the multisegment extracellular domain. This positions the integrin ligand binding domain in a membrane distal position allowing for enhanced interaction with ligands. CD28 consists of a single immunoglobulin-like extracellular domain that is unlikely to undergo large scale conformation changes, making the finding that it can increase its affinity in response to intracellular signaling all the more surprising.

In this report we have measured CD28 ligand binding using a cell adhesion frequency assay and show that TCR signaling can rapidly increase the avidity of CD28–CD80 interactions. Furthermore, our molecular dynamic (MD) simulation and site-directed mutagenesis data support a model whereby the TCR-induced increase in CD28 avidity results from a reorientation of the CD28 dimer to allow for bivalent ligand binding.

## Materials and Methods

### Ethics Statement

This study was carried out in strict accordance with the recommendations in the Guide for the Care and Use of Laboratory Animals of the National Institutes of Health and with the approval of the Animal Care and Use Committee at the University of Rochester (Protocol Number 2002-159).

### T Cells

CD4-postive T cells were purified from WT or CD28-deficient DO11.10 TCR transgenic mice or from CTLA-4-deficient, RAG-deficient, 5C.C7 TCR transgenic mice (Taconic) and activated in vitro with antigenic peptide and irradiated spleen cells. To limit induction of CTLA4 expression, in some experiments WT and CD28KO T cells were primed with CD80/CD86-negative transfectants (ProAd-ICAM). T cells were used 7–10 days after priming (14–21 days for CTLA-4-deficient T cells).

### Retroviral Infection

The K118I/K120P mutation was introduced into murine CD28 by overlapping PCR and confirmed by DNA sequencing. Both WT and K118I/K120P CD28 were fused at the C-terminus to monomeric YFP with a 4 amino acid linker (RSTG) as described [11] and cloned into the Murine Stem Cell Virus retroviral vector, MIGR1 (which was deleted for the IRES-GFP). The plasmid constructs were transiently transfected into the Phoenix Ecotropic packaging cell line (provide by G. Nolan, Stanford University, Palo Alto, CA) and virus in the supernatant was concentrated by PEG precipitation (retro-X, Clontech). CD28-deficient T cells were stimulated for 36 hours, isolated on a ficoll gradient, and transduced with retrovirus in the presence of 4 µg/ml polybrene by centrifugation at 2000 rpm (Rcf 670) for 60 minutes. Expression of YFP and CD28 were determined by flow cytometry 6–10 days after initial activation. WT and K118I/K120P CD28-YFP-positive T cells were purified by flow cytometry.

### Cell Conjugation and Immunofluorescence Microscopy

B7-negative (ProAdICAM-1) and B7-positive (ProAdICAM-1/B7-1) transfectants [11] were pre-incubated with or without 2.0 µg/ml of OVA peptide for 1 hour at 37°C, mixed with T cells at a 1∶1 ratio, and centrifuged at Rcf 2000 for 20 seconds at RT. The cell pellet was incubated for 5 minutes at 37°C. T cells were resuspended in 200 µl DMEM, plated on poly-L-lysine (Sigma, St. Louis, MO) coated coverslips for 3 minutes at 37°C, and fixed in 3% (w/v) paraformaldehyde. Samples were imaged at room temperature in Mowiol-DAPCO on a Zeiss Axiovert microscope with a 63×1.4 NA on a Plan-Apochromat oil immersion objective. Images were collected utilizing a CoolSNAP HQ monochrome CCD camera (Roper Scientific). Nearest-Neighbor deconvolution and digital analysis were performed using SlideBook software (Intelligent Imaging Innovations).

### Micropipet Cell Adhesion Assay

Streptavidin-coupled dynabeads (Invitrogen) were coated with 125 ng/ml biotinylated mouse-anti-human IgG1 (BD Phamingen) and used to capture 50–250 ng/ml mouse CD80-hIgG1Fc fusion protein (R&D). The beads were blocked with 1.0 µg/ml mouse uPAR-hIgG1Fc fusion protein (R&D) and the level of CD80 bound (100–500 molecules/µm2) was determined by staining with anti-CD80-PE and comparison to QuantiBrite beads (BD Pharmingen). For TCR stimulation, 1.0 µg/ml anti-CD3 (2C11) was bound to latex beads. The adhesion frequency of T cells to CD80-beads was determined at room temperature essentially as described [17], [18], [19]. Briefly, a previously activated, resting T cell was brought into contact with a CD80-coated bead using micromanipulators for 2 sec and then separated. Adhesion was scored by visual distortion of the T cell membrane upon dissociation. Contact was repeated 25 times for each individual T cell. The T cell was then brought into contact with an anti-CD3 coated bead and another 25 impingements with anti-CD28 beads were analyzed.

### Model Building and MD Simulations

The extracellular domain of CD28 is connected to the transmembrane domain by a 16-residue fragment (GKHLCPSPLFP), which was not included in the crystal structure of CD28 (PDB ID 1YJD). We manually added this segment to the crystal structure using the Build tool in pymol [20] and oriented this segment to generate a disulfide-linked dimer using the editing tool in pymol. MD simulations were carried out with the AMBER force field [21]. Three independent trajectories were carried out for each system to improve MD sampling and provide a way of assessing errors [22]. The molecules were neutralized by adding Cl-atoms and solvated in a truncated octahedron box. The size of the box was set to assure an 8 Å distance between the molecule and the edge of the box. Initial conformations were minimized as follows: 1) the protein was held fixed, while the solvent was minimized by 500 steps of steepest descent followed by another 500 steps of conjugate gradient; 2) the whole system was minimized by 1,000 steps of steepest descent followed by another 1,000 steps of conjugate gradient. After minimization, MD simulations were carried out using the ff99SB [23] force field and the TIP3P water model [24] in the NPT ensemble. The temperature was held constant at 300 K using the Langevin thermostat with a collision frequency g = 1.0ps^−1^. Hydrogen bonds were constrained using the SHAKE algorithm, [25], [26] which allowed a time step of dt = 2 fs. We used periodic boundary conditions with the Particle Mesh Ewald method [27] with a 9 Å cutoff to calculate long range electrostatic forces. The system was initially allowed to equilibrate at constant temperature for 50 ps without pressure coupling. After this, the pressure was kept constant at 1 atm by means of the Berendsen barostat [28]. Trajectories were analyzed using the cpptraj program from the AMBER suite. This includes calculations of distances, RMSD’s and buried surface areas. When calculating the RMSD with respect to the initial conformation, the stalk region was not included.

Binding of CD80 to WT CD28, K118I/K120P CD28 was modeled based on the binding mode predicted for CD80 and CTLA-4 [29]. To test whether this binding mode was also stable for CD28, A 41 ns MD simulation of a CD28 monomer bound to a CD80 monomer was carried out. The simulation was stable with RMSD values below 5Å (data not shown). To dock the CD80 molecules onto the simulated conformations, we used pymol’s alignment tool.

## Results

### TCR Signaling Can Enhance the Avidity of CD28–CD80 Binding

**Figure 1:**
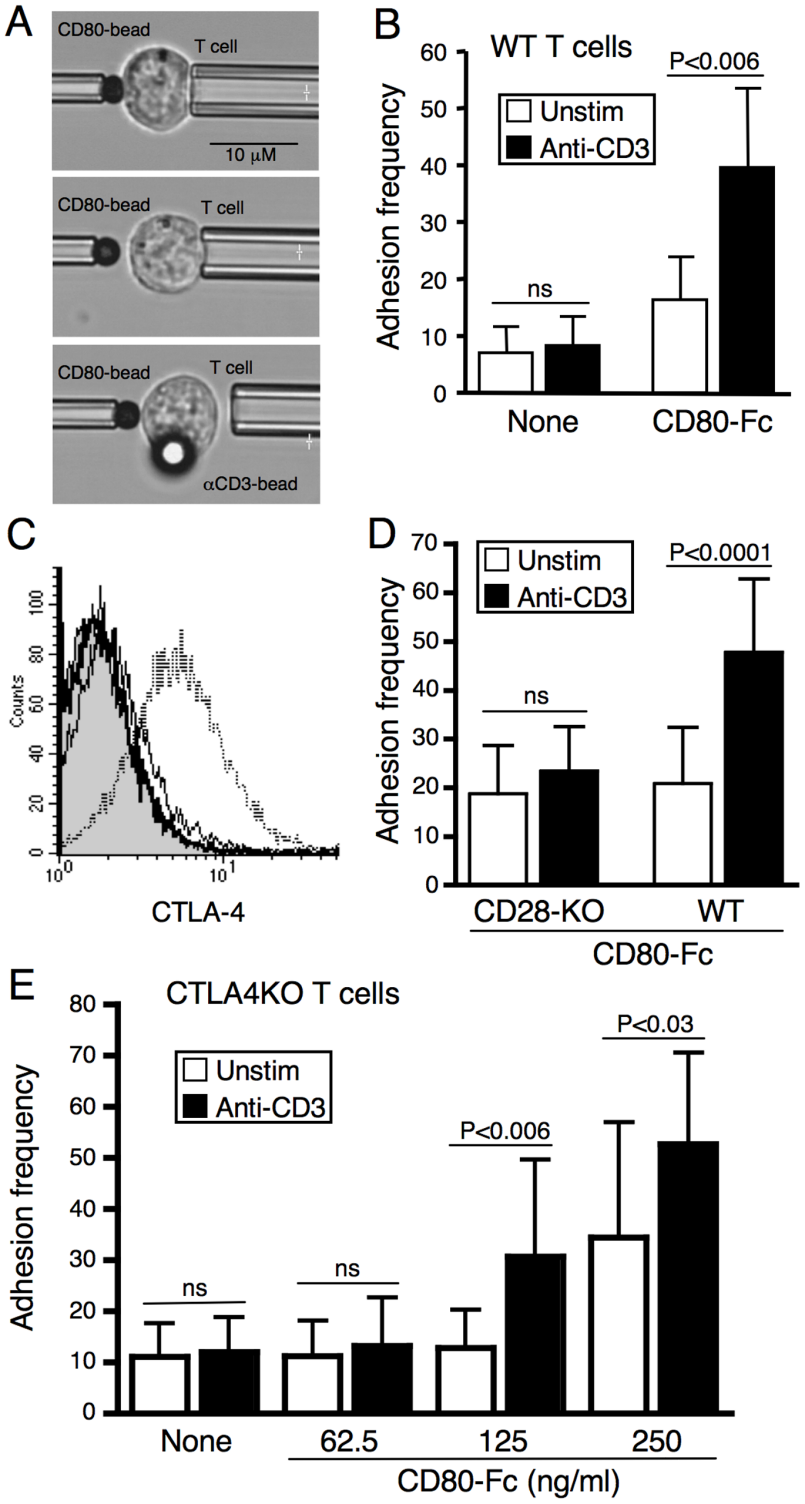
TCR signaling can enhance the avidity of CD28 ligand binding. (**A**) Images from a cell adhesion assay. Top, a resting T cell and CD80-coated bead brought into contact using micropipets. Middle, the same T cell 30s later after the cell and bead were separated. No adhesion was observed. Bottom, image of a T cell bound to an anti-CD3 coated bead, which is out of the plane of focus, but clearly distal to the site of interaction of the T cell with the CD80-bead. This image was taken shortly after the T cell was pulled away from the CD80-bead. In this case, the T cell-CD80-bead adhesion has caused the T cell to dislodge from the micropipet. (**B**) Cell frequency adhesion of in vitro primed and resting WT DO11.10 T cells to control beads (None) or beads coated with CD80 (CD80-Fc) in the absence (Unstim, empty bars) and after (Anti-CD3; filled bars) stimulation. No increase in adhesion to CD80 was detected when cells were stimulated with beads coated with anti-MHC class I (not shown). (**C** and **D**) WT and CD28-deficient T cells were primed with CD80/CD86-negative APC to reduce upregulation of CTLA-4. (**C**) Flow cytometry of CTLA-4 expression on WT (thin line) and CD28-deficient (thick line) T cells after in vitro priming. Isotype control (filled) and WT T cells primed in the presence of CD28 costimulation (dashed line) are included as negative and positive controls. (**D**) Cell frequency adhesion of WT and CD28-deficient (CD28KO) T cells that were primed with CD80/CD86-negative cells, to CD80-beads in the absence (Unstim) and after (Anti-CD3) stimulation. (**E**) Adhesion frequency of WT and CTLA-4-deficient (CTLA-4KO) T cells to control beads (None) or beads coated with increasing concentrations of CD80 in the absence (Unstim) and after (Anti-CD3) stimulation. Cell adhesion data are presented as mean ± SD of the adhesion frequency for individual cells (25 impingements each; n = 9–10, except for 62.5 ng/ml CD80-Fc in panel E, n = 6; p values for t tests between samples are shown; ns, not significant).

To determine whether TCR could enhance CD28–CD80 interactions we used a cell frequency adhesion assay [17], [18], [19], [30]. In this assay T cells are captured on a micropipet and repeatedly brought into contact with beads coated with CD80 (Figure 1A). Adhesion is detected by visual distortion of the T cell membrane during separation of the T cell-bead contact. The frequency of adhesion events is determined by the concentration of CD28 on the T cell surface, the concentration of CD80 on the bead, and the avidity of the CD28–CD80 interaction. A base line of adhesion was first established in the absence of TCR signaling, the T cell was then brought into contact with an anti-CD3 coated bead and the adhesion frequency to CD80-beads was measured. As shown in the bottom panel of Figure 1A, the T cell interaction site with the anti-CD3-bead is distal from the interaction site with the CD80-coated bead. Thus, any impact of TCR engagement is not mediated by local effects of CD3 cross-linking in the plasma membrane. Wild type (WT) T cells show a clear increase in adhesion frequency after stimulation with anti-CD3 (Figure 1B).

To confirm that this enhanced adhesion was mediated through CD28, we compared WT and CD28-deficient T cells. To limit any contribution of CTLA-4 to the adhesion assay, WT and CD28-deficient T cells were primed in the absence of CD28 costimulation to limit the induction of CTLA-4 expression (Figure 1C) [31]. Enhanced adhesion to CD80-beads after TCR signaling was only detected in WT cells, indicating that this adhesion was mediated by CD28 (Figure 1D). To further exclude a potential contribution of CTLA-4, we found that TCR signaling of CTLA4-deficient T cells could enhance binding to CD80-beads (Figure 1E). Taken together, these data indicate that TCR signaling can increase the avidity of CD28–CD80 interactions.

### Lysines at the Base of the CD28 Dimer Interface Control the Valency of CD28 Ligand Binding

**Figure 2:**
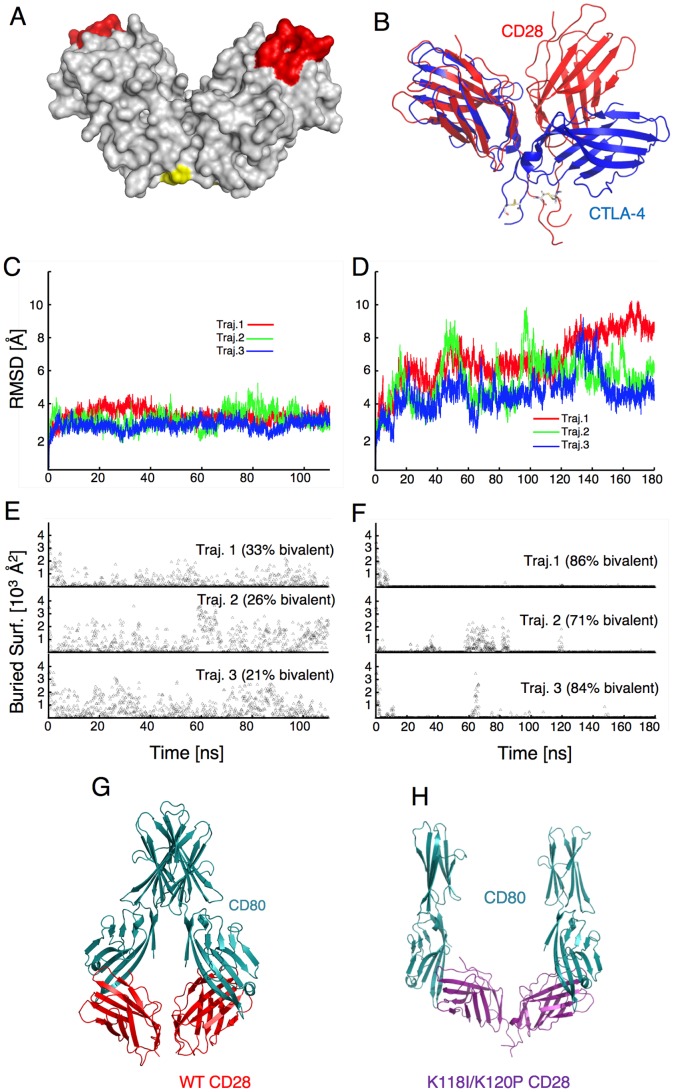
K118I/K120P CD28 rapidly adopts a conformation that would allow bivalent binding in molecular dynamic simulations. (**A**) Structural model of the extracellular domains of the CD28 homodimer illustrating the location of the ligand binding sites (red) and K118/K120 (yellow) at the base of the dimer interface. The K118/K120 residues are only visible on the left hand side monomeric unit in this view. (**B**) The structural model of CD28 (red) is superimposed on that of CTLA-4 (blue). The illustration shows that the monomeric units are structurally similar, but when forming the dimer, their relative orientations are different. Additional residues including the interchain di-sulfide bond were added at the end of the CD28 structural model shown (see methods for details). (**C** and **D**) Three independent trajectories were simulated of WT CD28 (C) or K118I/K120P CD28 (**D**) with the crystal structure of CD28 as the initial conformation and the RMSD with respect to the initial conformation are shown over time. For simulations of K118I/K120P CD28 (**D**), there is considerable rearrangement of the subunits at the beginning of the simulations, reflected by an increase in the RMSD. For trajectories 2 and 3, RMSD values start to stabilize towards the end of the simulation. (**E** and **F**) CD80 molecules were docked onto the simulated WT CD28 dimers (**E**) and K118I/K120P CD28 dimers (**F**), to obtain the corresponding CD28+ CD80 complexes. The surface area buried between carboxy-terminal domains of the docked ligands was calculated at various times. A value of zero indicates no buried surface and thus no contact between the ligands, which would allow for bivalent ligand binding. The fraction of bivalent-competent conformations along each of the three independent trajectories is indicated and the average over all three trajectories was 26% for WT CD28 and 81% for K118I/K120P CD28. (**G**) A representative conformation from simulations of WT CD28 (red) showing docked CD80 ligands (cyan). It can be seen that the orientation of the ligand binding sites precludes bivalent binding due to steric interference at the distal end of CD80. (**H**) A representative conformation from simulations of K118I/K120P CD28 (purple) showing bivalently-bound CD80 ligands (cyan).

One possible mechanism that could account for increased CD28 ligand binding is a change in valency. CD28 is a homodimer that contains two identical functional ligand-binding sites. Yet, soluble recombinant CD28 can only interact with ligand monovalently [32]. Evans et al. [33] obtained a crystal structure of monomeric CD28 (PDB ID 1YJD ) in complex with the Fab fragment from the 5.11A1 mitogenic antibody [34]. Although the CD28-Fab complex did not dimerize, a model for the CD28 dimer was proposed based on the lattice contacts in the crystal. The model is shown in Figure 2A. The structure of the monomer is very similar to that of CTLA-4, [29], [35], [36] and the CD28 dimer presents the same v-type topology of CTLA-4. However, superposition of the two homodimers (Figure 2B) shows that CD28 adopts a more compact conformation. This difference in orientation between the subunits in the CD28 and CTLA-4 dimers is significant because it is thought to control the valency of ligand binding. Although both ligand-binding sites are open in CD28, the CD28 ligands, CD80 and CD86, are elongated structures and steric interference distal to the ligand-binding site is thought to preclude bivalent binding [33]. In contrast, the dimer interface in CTLA-4 creates a greater angle between the ligand binding sites eliminating the steric interference that prevents bivalent binding in CD28. This difference in valency between these structurally related molecules raises the possibility that in the context of the cell membrane, TCR signaling could induce a change in the orientation of the CD28 monomers within the homodimer, that would allow for bivalent binding. This change in valency could then result in a functionally significant increase in CD28 avidity that would regulate CD28 ligand binding.

To determine whether enhanced CD28 ligand binding could be mediated through a reorientation of the monomers to allow for bivalent binding, we modified the dimer interface. Based on the crystal structure of CD28, it was proposed that the presence of two charged residues (K118 and K120, see Figure 2A) at the base of the dimer interface prevents CD28 from adopting a CTLA-4-like orientation [33]. Charge repulsion between these residues is thought to drive the base of the interface apart and, consequently, position the membrane distal ligand binding sites closer together. In CTLA4 the corresponding residues are hydrophobic (isoleucine and proline) and are thought to stabilize the base of the dimer interface, allowing the ligand binding sites to separate. To determine the impact of these two lysine residues in the orientation of the CD28 monomeric units, we used molecular dynamic (MD) simulations to study the effect of mutating these lysine residues to the corresponding hydrophobic residues in CTLA-4. Three independent simulations were carried out for the WT CD28 dimer and for the K118I/K120P mutant, with the crystal structure of CD28 as the initial conformation. For WT CD28 molecules, the dimer model is stable and the molecules largely remain in compact structures and the root mean square deviation (RMSD) with respect to the initial conformation remains below 5 Å (Figure 2C). In contrast, K118I/K120P CD28 does not remain in the initial conformation, as evidenced by a large initial increase in RMSD (Figure 2D). The structures eventually stabilize, although continued fluctuations in RMSD in the trajectories indicate that a final stable structure was not reached in the simulations. During the simulations, K118I/K120P CD28 molecules quickly undergo conformational changes that bring the 118–120 regions of the two monomers together, disrupting the initial orientation, causing the ligand binding regions to separate.

To assess whether the molecules populate conformations that would allow bivalent binding, CD80 molecules were docked onto the CD28 dimers, based on the ligand interface in the CD80-CTLA-4 co-crystals [29]. The buried surface between the carboxy-terminal domains of CD80 was calculated to measure the extent of steric interference that would preclude bivalent binding. The WT CD28 simulations generate large buried surfaces indicating that the ligands would often physically collide, indicative of monovalent conformations (Figure 2E). An illustration of this steric interference that precludes bivalent binding is shown in Figure 2G. In contrast, 81% of K118I/K120P CD28 conformations result in no buried surface between the CD80 molecules (Figure 2F), indicating that these conformations could bind ligand bivalently. An example of such conformation is shown in Figure 2H. This gain-in-function mutation is distal to the ligand-binding site and MD simulations do not predict any changes at the binding site itself that would suggest a change in affinity. Rather the MD simulations predict that K118I/K120P CD28 adopts a stable conformation that would accommodate bivalent binding. These data suggest that a change in valency is sufficient to provide a functionally relevant increase in ligand interactions.

### K118I/K120P CD28 Can Bind CD80 in the Absence of TCR Signaling

**Figure 3:**
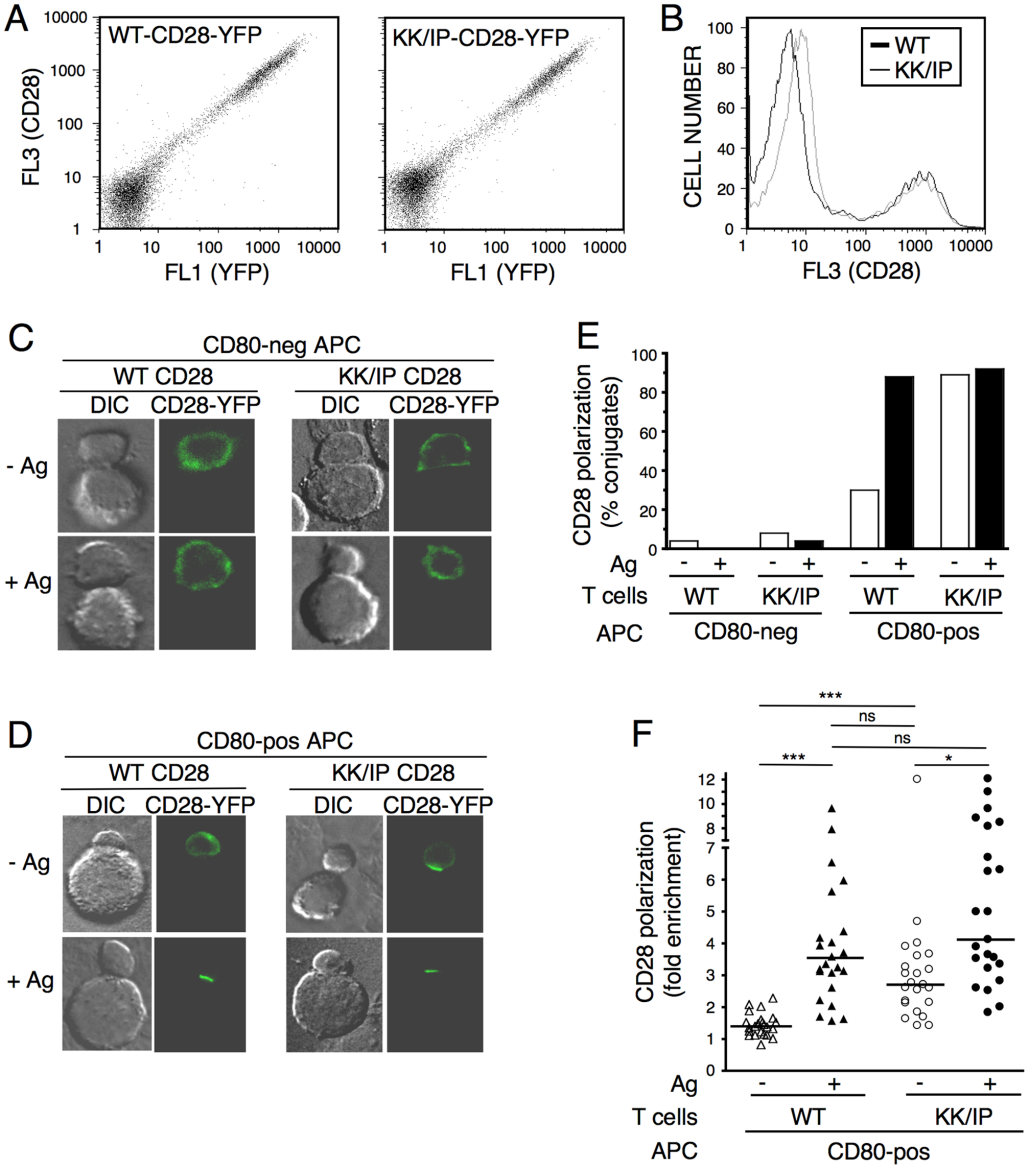
K118I/K120P CD28 is polarized toward CD80-positive cells in the absence of TCR signaling. (**A** and **B**) CD28-deficient, DO11.10 CD4 T cells were retrovirally transduced with WT or K118I/K120P CD28 fused to YFP, stained with anti-CD28 and analyzed by flow cytometry. Two color display (**A**) shows total transduced protein expression as detected by YFP (FL1, x-axis) and cell surface expression of CD28 as detected by anti-CD28 staining (FL3, y-axis). Single color display (**B**) shows relative cell surface expression of WT CD28 (thick line) and K118I/K120P (KK/IP) CD28 (thin line). (**C** and **D**) Representative images of WT and K118I/K120P CD28-YFP localization in cell:cell conjugates with CD80-negative (**C**) and CD80-positive (**D**) APC in the presence (+Ag) and absence (-Ag) of TCR signaling. DIC (digital image correlation) and fluorescent images are shown. (**E**) Individual conjugates were visually scored for CD28 polarization toward the interacting APC and the percentage of conjugates displaying polarized CD28 is shown (n = 25–28). (**F**) The efficiency of CD28 recruitment to CD80-positive APC was calculated by determining the ratio of YFP fluorescence within the T cell:APC contact site to the YFP fluorescence in the T cell plasma membrane distal to the contact site. Values for individual cells, population medians and statistical analysis (non-parametric Kruskal-Wallis ANOVA with Dunn’s multiple comparison) are shown (ns, not significant; * p<0.05; ***p<0.001).

To assess the impact of potential bivalent binding on CD28 ligand interactions, the K118I/K120P mutations were introduced into CD28 by site-directed mutagenesis. WT and mutated CD28 were fused to YFP at the C terminus and retrovirally transduced into CD28-deficient T cells. Both WT and K118I/K120P CD28 were expressed at equivalent levels at the cell surface (Figure 3 A and B). Importantly, the relative ratio of cell surface CD28 (as measured by antibody staining) to total CD28 (as measured by YFP fluorescence) was equivalent, indicating that the mutations did not impact the efficiency of CD28 expression at the plasma membrane (Figure 3A). To assess the ability of CD28 to interact with ligand, cell:cell conjugates were formed between T cells and APCs, and the localization of CD28 was determined by fluorescence microscopy (Figure 3 C–F). Neither WT nor K118I/K120P CD28 was polarized toward CD80-negative APC in the presence or absence of TCR signaling (Figure 3 C and E), indicating that CD28 polarization to the immunological synapse was dependent on ligand binding. For WT CD28, CD28-ligand binding requires concurrent TCR signaling [11] and WT CD28 is polarized toward CD80-positve APC only in the presence of antigen (Ag) (Figure 3 D–F). In contrast, K118I/K120P CD28 is polarized toward cells expressing CD80 in the presence and absence of Ag (Figure 3 D–F). Thus, mutation of the two lysine residues bypasses the apparent need for activation of ligand binding, allowing K118I/K1210P CD28 to be efficiently recruited towards the immunological synapse in the absence of TCR signaling.

**Figure 4:**
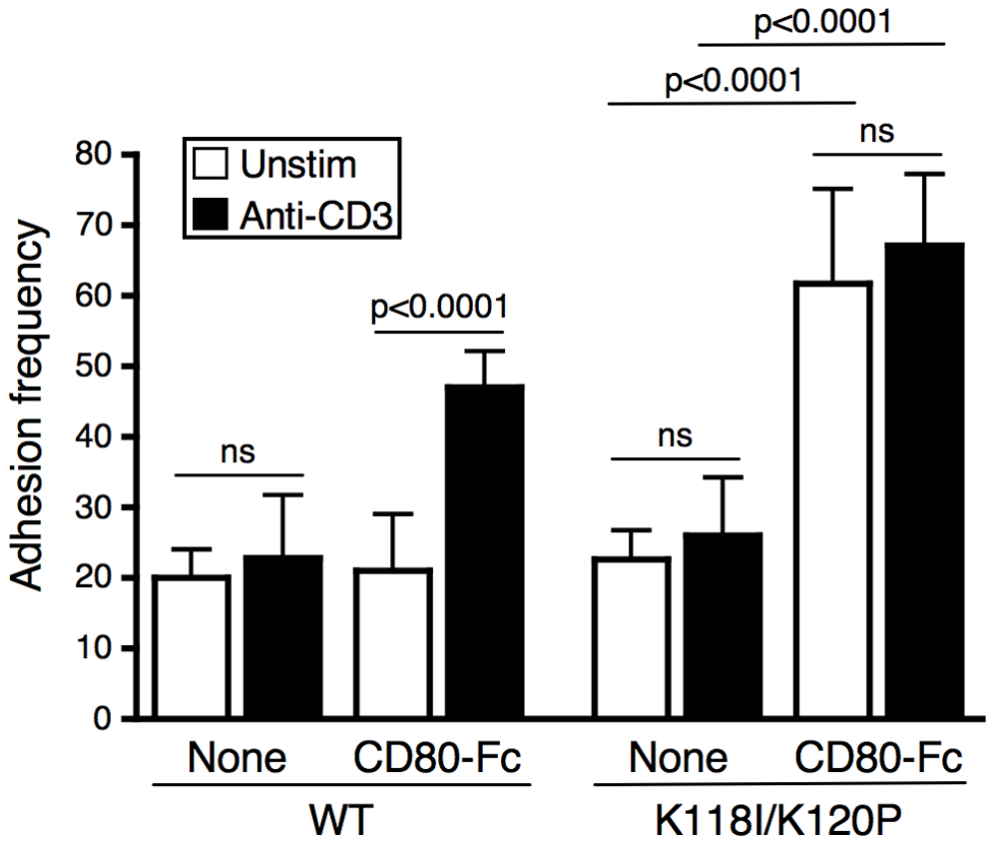
K118I/K120P CD28 binds CD80 with high avidity in the absence of TCR signaling. Cell frequency adhesion of CD28-deficient, DO11.10 T cells retrovirally transduced with WT CD28-YFP or K118I/K120P CD28-YFP to control beads (None) or beads coated with CD80 in the absence (Unstim) and after (Anti-CD3) stimulation. Cell adhesion data are presented as mean ± SD of the adhesion frequency for individual cells (25 impingements each; n = 6–7 for control beads and 12–14 for CD80-Fc beads; p values for t tests between samples are shown).

To confirm that the TCR-independent polarization of K118I/K120P CD28 to CD80-positve cells is mediated through enhanced ligand binding, we used the cell adhesion frequency assay (Figure 4). CD28-deficient T cells transduced with WT CD28 showed the same dependence on TCR signaling for adhesion to CD80-beads as seen in Figure 1 for T cells expressing endogenous CD28. In contrast, CD28-deficient T cells transduced with K118I/K120P CD28 showed significant binding to CD80-beads even in the absence of TCR signaling (Figure 4). These results, together with the simulations showing that K118I/K120P CD28 adopts bivalent conformations support the model that CD28 ligand interactions can be regulated by a change in valency.

### WT CD28 can Adopt Conformations that Allow for Bivalent Binding

**Figure 5:**
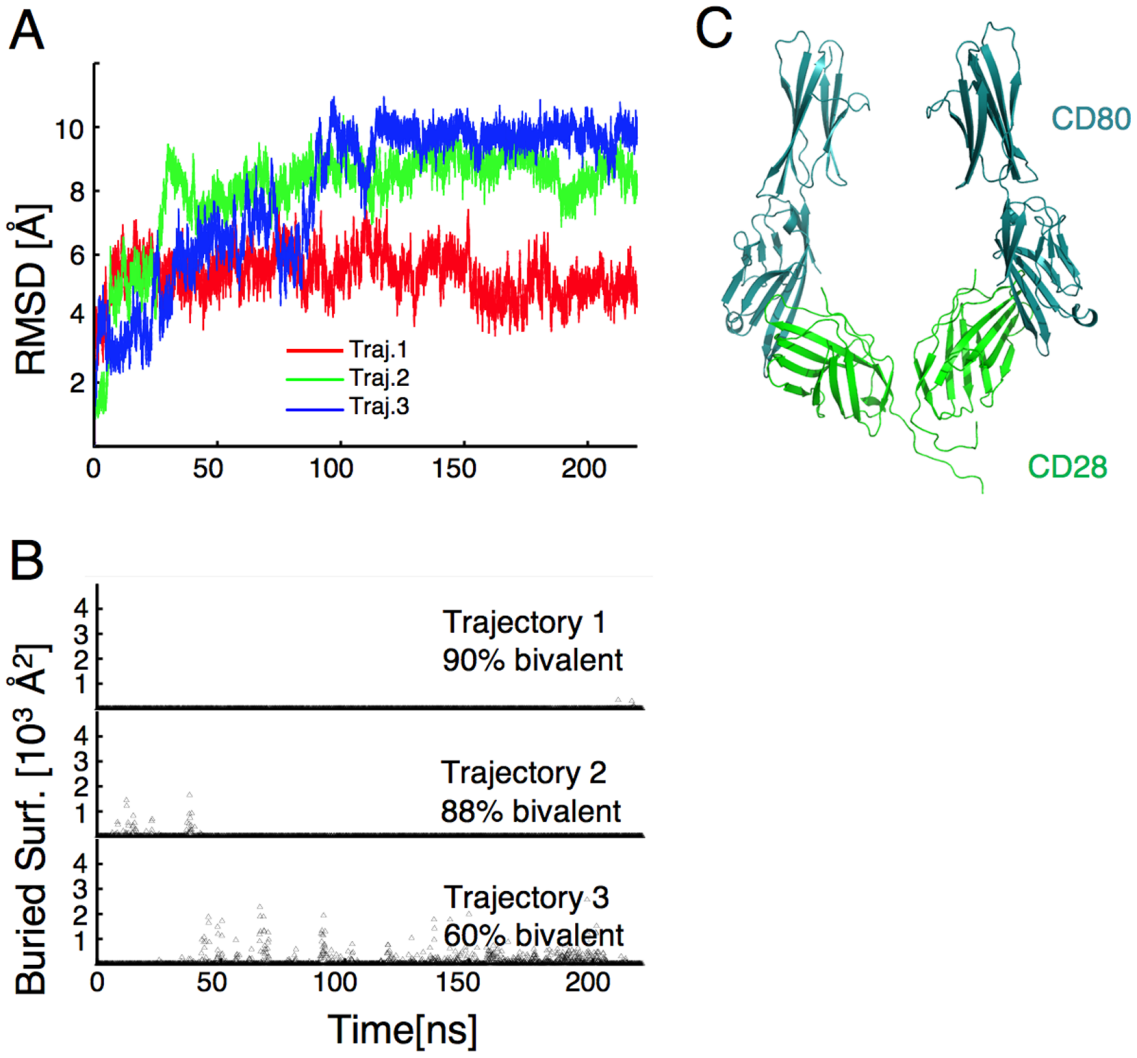
WT CD28 adopts a bivalent binding conformation when starting from the CTLA-4 dimer orientation. MD simulations were run with WT CD28 starting from a CTLA-4 dimer orientation. (**A**) The RMSD of three independent trajectories with respect to the initial conformation are shown over time. There is considerable rearrangement of the subunits at the beginning of the simulations, reflected by an increase in the RMSD. The conformational fluctuations stabilize after the first 100 ns of simulation, although the trajectories adopt different conformations. This instability was not inherent to this dimer orientation, as the RMSD of MD simulations of CTLA-4 remained below 4Å (data not shown). (**B**) CD80 molecules were docked onto the simulated CD28 dimers to obtain the corresponding CD28-CD80 complexes. The surface area buried between the docked ligands was calculated at various times along each of the three independent trajectories to estimate the fraction of bivalent-competent conformations; the average over all three trajectories was 79%. (**C**) A representative conformation of CD28 (green) showing docked CD80 ligands (cyan) illustrates the potential for bivalent ligand binding.

If TCR inside-out signaling is regulating CD28 ligand binding through a change in valency, then WT CD28 must be able to adopt a dimer orientation that would accommodate bivalent ligand binding. To assess this possibility, we carried out MD simulations of CD28 with the dimer orientation of CTLA-4 (see Figure 2B) as the initial conformation. We used the CD28 crystal structure [33] to build the monomers, but they were superimposed on the model of a CTLA-4 dimer (PDB ID 3OSK) [37], resulting in a molecule with a greater distance between the ligand binding sites. The initial conformation used in these simulations is not stable and there is considerable rearrangement in the orientation of the monomers at the beginning of the simulation, as evidenced by a increase in the RMSD (Figure 5A). However, it should be noted that no restraints have been imposed on the C-termini connecting the extracellular and transmembrane domains. Such a restraint could contribute to stabilization of bivalent conformations. Nevertheless, in all trajectories, the molecule primarily populates bivalent conformations. When we docked CD80 molecules on the CD28 conformations we found that, on average, 79% of conformations can accommodate two ligands (Figure 5B). These conformations did not resemble that of CTLA-4, where the orientation of the subunits is such that the ligands lie parallel to each other. Instead, in our simulations, bivalency was achieved by a slight rotation of the CD28 monomers around the dimer interface. When the CD80 ligands were docked onto CD28, the CD80 molecules formed an angle with each other placing their distal ends far enough apart to allow for bivalent binding (Figure 5C).

**Figure 6:**
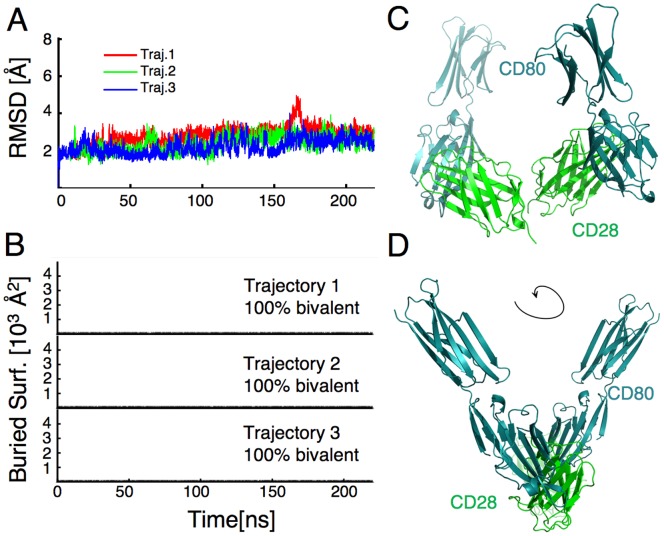
WT CD28 can adopt a stable conformation that would allow bivalent binding. MD simulations were run with WT CD28 starting from a rotated dimer orientation predicted from the simulations starting from the CTLA-4 dimer orientation. (**A**) The RMSD with respect to the initial conformation for three independent trajectories indicate that these dimer conformers are very stable. (**B**) When CD80 molecules were docked onto the conformations, there was no surface buried between the ligands, indicating that all conformations were bivalent. A representative bivalent conformation of CD28 (green) with docked CD80 molecules (cyan) is shown to illustrate the CD28 dimer (**C**) and in a rotated view to show the orientation of the docked CD80 molecules (**D**).

Therefore, we designed a new initial conformation in which the subunits of CD28 were rotated with respect to their orientation in the crystal structure in the fashion observed in the simulations starting from the CTLA-4 dimer orientation. Care was taken that, despite the rotation in the initial conformation, the hydrophobic residues that formed the dimer interface in the crystal structure were still part of the interface. This new initial conformation was stable, as indicated by RMSDs below 5Å for three independent simulations (Figure 6A). The small fluctuations observed during the simulations produced only bivalent conformations (Figure 6B), as in the example shown Figure 6C. Interestingly, because of the rotation of the dimer interface the CD80 molecules come off at a more oblique angle as seen in the rotated view in Figure 6D. These results suggest that in the presence of an appropriate external force, that might be initiated by a TCR-induced reorganization of the CD28 cytosolic domains, the WT CD28 dimer can form stable dimer interfaces that position the ligand binding sites in a spatial orientation that allows for bivalent interactions with CD80.

## Discussion

In this report we show that TCR signaling can increase the avidity of CD28 ligand binding. These results are consistent with previous studies showing that TCR signaling can initiate CD28 localization at the immunological synapse [14] and that sustained TCR signaling is required to maintain CD28 at the immunological synapse [11], [13]. Taken together, the results indicate that the ability of TCR signaling to increase the 2-dimensional avidity of CD28 ligand interactions would drive the effective recruitment of CD28 to the immunological synapse and ultimately the transmission of functional costimulatory signals from CD28 that are required for T cell activation. Traditionally we think of CD28 as a regulator of TCR signaling and most models of TCR/CD28 signal integration take place during downstream signaling events. Our results create a new paradigm for this process indicating that TCR can actually modify CD28 function and that integration of TCR and CD28 signaling may be initiated through regulation of ligand binding events at the plasma membrane. These results imply that TCR signaling may regulate the ability of both the primary costimulatory molecule, CD28, and the primary adhesion molecule, LFA-1, to engage their respective ligands. Thus, initial TCR signaling can coordinate LFA-1 and CD28 ligand engagement during immunological synapse formation, providing new insights into how coincidence detection of antigen recognition in the context of costimulation regulates the initiation of T cell immune responses and determines the fate of T cells following initial encounter with antigen.

Regulation of receptor-ligand interactions through this inside-out receptor cross-talk provides an interesting paradigm for the sequential receptor activation events that control complex cellular processes. This pathway has been extensively characterized for integrins, including signaling from the activating receptors, modifications and structural changes within the cytosolic domains, association with regulatory proteins, and the conformational changes that occur within the extracellular domain to enhance integrin ligand binding [15], [16]. Inside-out signaling has also been implicated in regulating ligand binding for cadherin [38] and L1 [39] adhesion receptors and differential reactivity to monoclonal antibodies supports a change in the orientation of the multiple extracellular domains. Cytokine signaling has been shown to enhance ligand binding to the IgA and IgG Fc receptors [40], [41]. The signaling pathways that mediate this effect have been identified [42], [43], but the changes to the extracellular domains to allow for enhanced ligand binding remain unknown. Interesting for CEACAM1 [44], interactions between GXXXG transmembrane motifs appears to stabilize cis-dimers in the inactive form. Calmodulin binding to the cytosolic domains disrupts the dimers, allow the monomeric CEACAM1 molecules to form trans-interactions and cell adhesion with neighboring cells. Our results indicate that CD28 is also regulated through inside-out signaling supporting the possibility that inside-out regulation of receptor-ligand interactions may be a general mechanism for receptor cross-talk at the cell surface.

In our micropipet adhesion experiments we have isolated TCR and CD28 ligands on separate surfaces (anti-CD3-coated and CD80-coated beads, respectively). Because these beads contact the T cell at discrete sites on the plasma membrane, it is unlikely that the ability of TCR signaling to enhance CD28 ligand binding is mediated by local changes within the plasma membrane (such as actin polymerization, lipid membrane domains, or receptor capping). Rather, it is more likely to be mediated by activation of specific downstream signaling pathways that can function at a distance. During T cell activation by antigen presenting cells, TCR and CD28 are colocalized within both microclusters and within the cSMAC of the mature immunological synapse. So it is possible that additional local effects of TCR engagement, or contributions of signaling from additional receptors, could further impact on the activation (or inactivation) of CD28-ligand binding.

Both CD28 and CTLA-4 can interact with the ligands, CD80 and CD86. The receptor-ligand binding interface is conserved and both ligands can form functional interactions with both receptors [33], [45]. However, the interactions between these four receptor-ligand pairs are not identical. CTLA-4 binds to both CD80 and CD86 with a higher affinity than CD28 and CD80 binds to both CTLA-4 and CD28 with a high affinity than CD86 (the hierarchy of interactions is: CTLA4:CD80> CTLA4:CD86> CD28:CD80> CD28:CD86) [32]. The relative difference in affinity of CTLA-4 to CD28 for binding to CD80 is 20-fold, whereas the difference in binding to CD86 is only 8-fold [32]. In a competitive environment (such as after CTLA-4 is induced on T cells), CTLA-4 could then out compete CD28 for binding to CD80 to a greater degree than binding to CD86. This predicted preference for binding in a competitive environment has been confirmed experimentally [46]. The kinetics of expression of these receptor-ligand pairs and ligand-specific blocking experiments have suggested that CD86 functions to promote T cell activation (consistent with the requirement for CD28 costimulation for T cell activation), while CD80 suppresses T cell activation (consistent with CTLA-4 mediated suppression). CD28 is constitutively expressed on naïve T cells, whereas CTLA-4 is only induced following activation [31]. Likewise, CD86 is constitutively expressed on APC and rapidly upregulated upon activation, whereas expression of CD80 on APC is kinetically delayed [47], [48]. Thus, during initial T cell priming when CD28 costimulation is required for activation, CD86 is the primary ligand available on APC. Later, in the response, when the magnitude of T cell activation needs to be tempered, the inhibitory receptor, CTLA-4, is induced on the T cells, coincident with the induction of CD80 expression on the APC. *In vivo* blocking experiments with CD80 or CD86-specific antibodies or with CD80/CD86 selective knockout models generally support this association of CD28 with CD86 and CTLA4 with CD80 (for example see [49], [50], [51], [52], [53], [54]). However, these findings remain controversial with other groups reporting role for CD80 in T cell activation and CD86 in immunosuppression (for example see [55], [56], [57]). Whether these differences in CD80 and CD86 function *in vivo* are related to preferential interactions with CD28 and CTLA-4 or whether they are also related to the selective activation of T effector subsets or T regulatory cells and/or to restricted expression of CD80 or CD86 within specific target tissues is not clear [45], [58]. In our studies we have shown that TCR can increase CD28 ligand binding to CD80 in the presence and absence of CTLA-4 expression. Given the very rapid off-rate of monomeric CD28 from CD86 and the predicted 100-fold increase in the stability of bivalent versus monovalent binding in solution [32], we would predict that impact of TCR-mediated activation of CD28 ligand binding would be more significant in interactions with CD86 than CD80.

Our results support a model whereby TCR signal regulates CD28 ligand binding through a change in valency within the CD28 dimer. Although CD28 is expressed as a disulfide linked dimer, recombinant soluble CD28 has been shown to only bind ligand monovalently [32]. In the crystallographic model, monovalency is determine by the orientation of the dimer interface. In contrast, the homologous receptor, CTLA-4, presents a different dimer interface and can bind bivalently [29], [32], [35], [36]. Therefore, we considered the possibility that TCR-mediated inside-out signaling would enhance CD28 ligand binding through a change in valency. Like integrins, CD28 activation would be initiated by a change in the orientation of the cytosolic tails in the dimer [14], but rather than inducing large scale conformational change in the lumenal domains like integrins, the rigid body immunoglobulin domains of CD28 would simply change their orientation in respect to one another, allowing for bivalent ligand binding and increased avidity. Consistent with this hypothesis, we have shown that disruption of a single ligand-binding site within the CD28 dimer, results in a significant decrease in recruitment to the immunological synapse, indicating that both ligand binding sites within a CD28 dimer are important for efficient ligand interaction [14].

To test this model more directly, we show here that introduction of two CTLA-4 residues at the base of the dimer interface of CD28 allows for enhanced ligand binding in the absence of TCR signaling. This gain-in-function mutation is distal to the ligand binding site and MD simulations do not predict any changes at the binding site itself that would suggest a change in affinity. Rather the MD simulations predict that K118I/K120P CD28 adopts a stable conformation that would accommodate bivalent binding. These data suggest that a change in valency is sufficient to provide a functionally relevant increase in ligand interactions. Although mutation of K118/K120 bypasses the need for TCR signaling for ligand binding, TCR signaling does result in increased localization of K118I/K120P CD28 to the immunological synapse (Figure 3F). It is possible that the K118I/K120P mutation does not fully mimic the dimer reorientation induced by TCR signaling or that additional factors can contribute to the organization of the mature immunological synapse. Nevertheless, our data do show that an extracellular conformational change in CD28 leads to increased avidity of the molecule and capping behavior commensurate with the natural activation response.

To determine whether WT CD28 could stably adopt a bivalent conformation we used MD simulations. We do not yet understand how TCR signals are transduced through the cytosolic domain to induce high avidity CD28 conformers, so we could not simulate this process precisely. Rather, we started the simulations with WT CD28 sequences in the dimer interface orientation of CTLA-4. This orientation was not stable, but WT CD28 did mostly reside in conformations that could interact with CD80 bivalently. Interestingly, the CD28 conformers that could bind bivalently adopted a rotated dimer orientation. When we started from this conformation, the MD simulations predicted a very stable orientation that remained bivalent. These data suggest that WT CD28 can form stable dimer interfaces that position the ligand binding sites in a spatial orientation that allows for bivalent interactions with CD80. We do not know what other constraints might be imposed by TCR-mediated inside-out signaling. Nevertheless, this result suggests that in the presence of an appropriate external force, that could be initiated by a TCR-induced reorganization of the CD28 cytosolic domains, the CD28 dimer can more favorably adopt a bivalent-binding conformation.

In recognition of the biological importance of CD28, a number of immunotherapeutics have been designed to target CD28. The most effective of these has been a soluble form of CTLA-4 (CTLA-4-Ig; Abatacept), which has been FDA approved to treat RA and may be useful in suppressing other autoimmune diseases [59], [60], [61]. Small molecule mimics of the CTLA-4 ligand-binding site have also been developed [62], [63], [64], [65], but both these and CTLA4-Ig target the CD80 and CD86 ligands and indiscriminately inhibit both CD28 and CTLA-4 binding and function. Our identification of a novel step in the regulation of CD28 activity provides a new conceptual framework on how signaling through this receptor is controlled. The proposed structurally distinct high and low avidity isoforms of CD28 could provide a new platform for isolation of CD28-specific immunotherapeutics.
